# Phylogenetic Position and Morphological Characteristics of the Plagiorchioid Trematode, *Skrjabinoplagiorchis polonicus* (Soltys, 1957), a Parasite of Rodents

**DOI:** 10.3390/biology14101423

**Published:** 2025-10-16

**Authors:** Alexander A. Kirillov, Nadezhda Y. Kirillova, Sergei V. Shchenkov

**Affiliations:** 1 Samara Federal Research Center of the Russian Academy of Sciences, Institute of Ecology of Volga River Basin of the Russian Academy of Sciences, Togliatti 445003, Russia; nadinkirillova2011@yandex.ru; 2 Department of Invertebrate Zoology, Saint Petersburg State University, St. Petersburg 199034, Russia; svshchenkov@yandex.ru

**Keywords:** digenea, Omphalometrinae, Plagiorchiidae, morphological features, molecular phylogeny, Volga region

## Abstract

**Simple Summary:**

The taxonomic position of the genus *Skrjabinoplagiorchis*, represented by the sole species *Skrjabinoplagiorchis polonicus*, remains unclear. Our paper presents the morphological and morphometric features of two trematode species parasitizing small mammals in the Middle Volga region (European Russia): *S. polonicus* from the liver of the herb field mouse (*Apodemus uralensis*) and *Rubenstrema exasperatum* from the small intestine of the common shrew (*Sorex araneus*). For the first time, we provide molecular data on these species of helminths in mammals from Russia. Our results establish the taxonomic affiliation of *S. polonicus* within the subfamily Omphalometrinae. Previously, parasitic flatworms of this subfamily were known only as parasites of insectivorous mammals belonging to the order Eulipotyphla.

**Abstract:**

The genus *Skrjabinoplagiorchis* is monotypic, with *Skrjabinoplagiorchis polonicus* parasitizing rodents in the Western Palaearctic. This genus is classified within the family Plagiorchiidae; however, its taxonomic position remains unclear. In the present study, two species of digeneans, *S. polonicus* and *Rubenstrema exasperatum*, found in small mammals in the Middle Volga region (European Russia), were examined. We provide morphological descriptions of the studied trematodes complemented with a molecular phylogenetic analysis of partial sequences of the 28S rDNA gene, obtained for these helminths of mammals in Russia for the first time. Based on morphological data and the results of molecular phylogenetic analysis, we reassign the genus *Skrjabinoplagiorchis* from the subfamily Plagiorchiinae to the subfamily Omphalometrinae. Previously, digeneans of the subfamily Omphalometrinae were known only as parasites of insectivores of the order Eulipotyphla.

## 1. Introduction

The trematode *Skrjabinoplagiorchis polonicus* (Sołtys, 1957) is the type-species of the genus *Skrjabinoplagiorchis* Petrov & Merkusheva, 1963 [1]. This species was first described by Soltys in 1957 as *Plagiorchis polonicus* from the common field vole, *Microtus agrestis* (Linnaeus, 1761), in Poland [2]. However, this trematode was earlier isolated by Schultz and Skworzow [3] in 1931 while describing *Plagiorchis arvicolae*. According to Sharpilo and Iskova [1], the material obtained by Schultz and Skwortsow [3] included two forms: *S. polonicus* and *P. arvicolae* proper. In 1963, Petrov and Merkusheva described *Skrjabinoplagiorchis vigisi* Petrov & Merkusheva, 1963 from the herb field mouse, *Apodemus uralensis* (Pallas, 1811), in Belarus and established the genus *Skrjabinoplagiorchis* as a separate lineage within the family Plagiorchiidae, with *S. vigisi* as the type-species [4].

Krasnolobova [5,6] removed *Plagiorchis polonicus* and *Plagiorchis ondatrae*, described by Andrejko [7] in 1965, from the genus *Plagiorchis* and transferred them to the genus *Skrjabinoplagiorchis*, as they align with its diagnosis. The main morphological differences between these two genera include the shape and position of the testes, the placement and extent of the uterus, and the site of infection within the host [1,5,6].

Genov [8] synonymized several species (*S. ondatrae* (=*P. ondatrae*), *Skrjabinoplagiorchis morosovi* Varenov, 1965, *Skrjabinoplagiorchis dogieli* Semenova, 1966, and *Skrjabinoplagiorchis obensis* Fedorov, 1976) [7,9,10,11] with *S. vigisi*. He noted the significant similarity between *S. vigisi* and *P. polonicus* and proposed transferring the latter to the genus *Skrjabinoplagiorchis*. Sharpilo and Iskova [1] agreed with the conclusions of Genov [8] and further reduced *S. vigisi* to a synonym of *S. polonicus*, establishing the latter as the type-species of the genus. They also considered the species *Skrjabinoplagiorchis skrjabini* (Kadenazii, 1960) to be a synonym of *S. polonicus* [12]. According to Sharpilo and Iskova [1], the genus *Skrjabinoplagiorchis* is monotypic, with *S. polonicus* parasitizing rodents in the Western Palaearctic.

The species *Rubenstrema exasperatum* (Rudolphi, 1819) was described by Rudolphi in 1819 as *Distomum exasperatum* based on material provided by Bremser from the Eurasian water shrew, *Neomys fodiens* (Pennant, 1771) [13]. Dollfus [14] established the subgenus *Rubenstrema* Dollfus, 1949 (within the genus *Opisthioglyphe* Looss, 1899). Skryabin and Antipin, in 1962 [15], raised the status to an independent genus within the family Omphalometridae, which includes parasites found only in Eulipotyphla. The survey of phylogenetic relationships among omphalometrids conducted by Tkach et al. [16] confirms the position of the genus *Rubenstrema* within this group and suggests that Omphalometrinae should be considered a subfamily within the family Plagiorhiidae. Further, Tkach [17] considers omphalometrids to be a separate family, taking into account the distinguishing morphological features of these trematodes.

In this study, we investigated *S. polonicus* and *R. exasperatum* from mammals in the Middle Volga region (European Russia). We used morphological and molecular genetic approaches to clarify their phylogenetic relationships and the taxonomic position of the genus *Skrjabinoplagiorchis*.

## 2. Materials and Methods

### 2.1. Material Collection and Morphological Examination

Sampling was conducted in the Mordovia Nature Reserve in 2022. A total of 28 trematodes were studied, including nine specimens of *S. polonicus* collected from three individuals of *A. uralensis*, and nineteen specimens of *R. exasperatum* collected from three individuals of the common shrew, *Sorex araneus* Linnaeus, 1758. The material for the parasitological study was obtained during the inventory of populations of small mammals. Counts were conducted regularly in accordance with the research topics of the Mordovia Nature Reserve, approved by the Ministry of Natural Resources and Ecology of Russia.

Only alive mature trematodes were collected for further research. For morphological examination, flukes were isolated from the liver (*S. polonicus*) and small intestine (*R. exasperatum*) of the hosts, rinsed in saline solution, and heat-killed in hot water. The samples were then stained with aceto-carmine, dehydrated in an ethanol series (70–96%), cleared in clove oil, and mounted in Canada balsam [18]. For molecular phylogenetic analysis, trematode specimens were fixed in 96% ethanol and stored at +4 °C.

Drawings were made using the Levenhuk M500 BASE Digital Camera and the RA-7 drawing tube (Lomo, Saint Petersburg, Russia) attached to the MBI-9 light microscope (Lomo, Saint Petersburg, Russia). All measurements are given in millimeters. Literature information for the comparative analysis was taken only from published works that contained drawings and morphometric data on *R. exasperatum* and *S. polonicus*. The digeneans were identified according to Skrjabin [15], Genov [8], Sharpilo and Iskova [1], and Kirillov et al. [19]. Voucher specimens are stored in the parasitological collection of the Institute of Ecology of the Volga River Basin of the Russian Academy of Sciences (Togliatti, Russia).

### 2.2. Molecular Data and Phylogenetic Analysis

Total DNA isolation and amplification were performed in full agreement with protocol of Kirillova et al. [20]. For amplification of D1-D3 region of 28S rDNA, forward primer 28sy (5′-CTA ACC AGG ATT CCC TCA GTA ACG GCG AGT-3′) and reverse primer 28sz (5′-AGA CTC CTT GGT CCG TGT TTC AAG AC-3′) [21] were used.

All amplicons were sequenced in “Evrogen” company (Moscow, Russia) using the same primer pair. Sequences from both forward and reverse primers were assembled using Chromas Pro v. 1.7.4 (Technelysium Pty Ltd., South Brisbane, Australia). Bayesian Inference (BI) analysis was performed on the 28S rDNA dataset (Supplementary Table S1). The final length of alignment was 1228 bp. The alignment includes all available sequences of Plagiorchiidae clades and closely related taxa. The best fitted evolutionary model for phylogenetic analysis was GTR + G + I, as estimated with MrModeltest v. 2.4 [22]. Bayesian analysis was performed using MrBayes v. 3.2.7a at local workstation for 15,000,000 generations. The quality of the chains was estimated using built-in MrBayes tools. Based on the estimates, the first 25,000 generations were discarded for burn-in. We included *Paragonimus miyazakii* (Kamo, Nishida, Hatsushika & Tomimura, 1961) (Paragonimidae) as an outgroup according to the recent phylogenetic reconstructions [23].

## 3. Results

### 3.1. Molecular Phylogenetic Analysis

**Figure 1:**
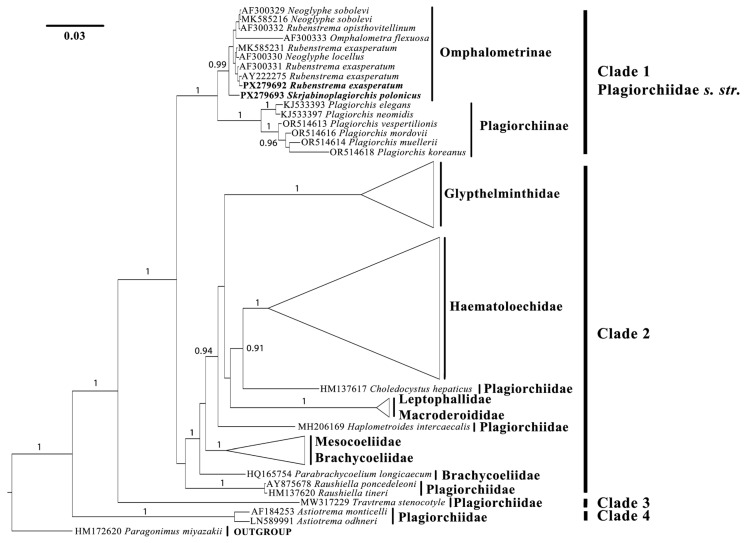
Results of molecular phylogenetic analysis (BI) based on partial 28S rDNA sequences. BI supports above 0.9 are indicated. Newly obtained sequences are shown in bold.

Based on the results of the molecular phylogenetic analysis, all 55 ingroup sequences clustered into four clades: Clades 1–4 (see Figure 1). The newly obtained sequence of *R. exasperatum* clusters together with other representatives of the genus within Clade 1 (Omphalometrinae subfam.). Within this cohesive subclade, the sequence AF300330 (*Neoglyphe locellus* (Kossack, 1910)) is also present. The sister group to this subclade is formed by two sequences of *Neoglyphe sobolevi* (Shaldybin, 1953) and a single sequence of *Rubenstrema opisthovitellinum* (Sołtys, 1954), which exhibit minimal nucleotide differences. The sequence AF300333 (*Omphalometra flexuosa* (Rudolphi, 1809)) clusters with *Neoglyphe* Shaldybin, 1954 and *Rubenstrema* without significant support. The sequence of *S. polonicus* occupies a basal position relative to these clades, with high posterior probability. All of the aforementioned sequences are grouped with a sister clade consisting of six sequences from the genus *Plagiorchis* Lühe, 1899 (family Plagiorchiidae), with full posterior probability (Figure 1).

Clade 2 encompasses multiple subclades, currently subdivided into several trematode families. A well-defined group of sequences comprises representatives of the genus *Glypthelmins* Stafford, 1905 (family Glypthelmintidae). This genus forms a sister clade to a group of several other genera. The genus *Haematoloechus* Looss, 1899 (family Haematoloechidae) constitutes a well-supported monophyletic clade. In relation to it, *Choledocystus hepaticus* (Lutz, 1928) occupies a basal position; this species is currently classified within the family Plagiorchiidae (Figure 1). Two representatives of the family Leptophallidae (*Leptophallus nigrovenosus* (Bellingham, 1844) and *Metaleptophallus gracillimus* (Lühe, 1909)) are closely related to a member of the family Macroderoididae (*Macrodera longicollis* (Abildgaard, 1788)). Additionally, another distinct position is occupied by *Haplometroides intercaecalis* Silva, Ferreira & Strüssmann, 2007, a representative of the family Plagiorchiidae. Most sequences of the family Brachicoeliidae form a monophyletic clade; however, one species—*Parabrachycoelium longicaecum* Perez-Ponce De Leon, Mendoza-Garfias, Razo-Mendivil & Parra-Olea, 2011, also currently classified within Brachicoeliidae—forms a separate clade, albeit with low Bayesian support. The most basal position within Clade 2 is occupied by sequences of the genus *Rauschiella* Babero, 1951, which is presently assigned to the family Plagiorchiidae (Figure 1).

Clade 3 is represented by a single sequence of *Travtrema stenocotyle* (Cohn, 1902) (currently within the family Plagiorchiidae), with full Bayesian support. Finally, the most basal Clade 4 includes two representatives of the genus *Astiotrema* Looss, 1900 (family Plagiorchiidae) (Figure 1).

### 3.2. Taxonomic Summary and Morphological Characteristics

Superfamily Plagiorchioidea Lühe, 1901

Family Plagiorchiidae Lühe, 1901

Subfamily Omphalometrinae Looss, 1899

Genus *Skrjabinoplagiorchis* Petrov & Merkusheva, 1963

**Figure 2:**
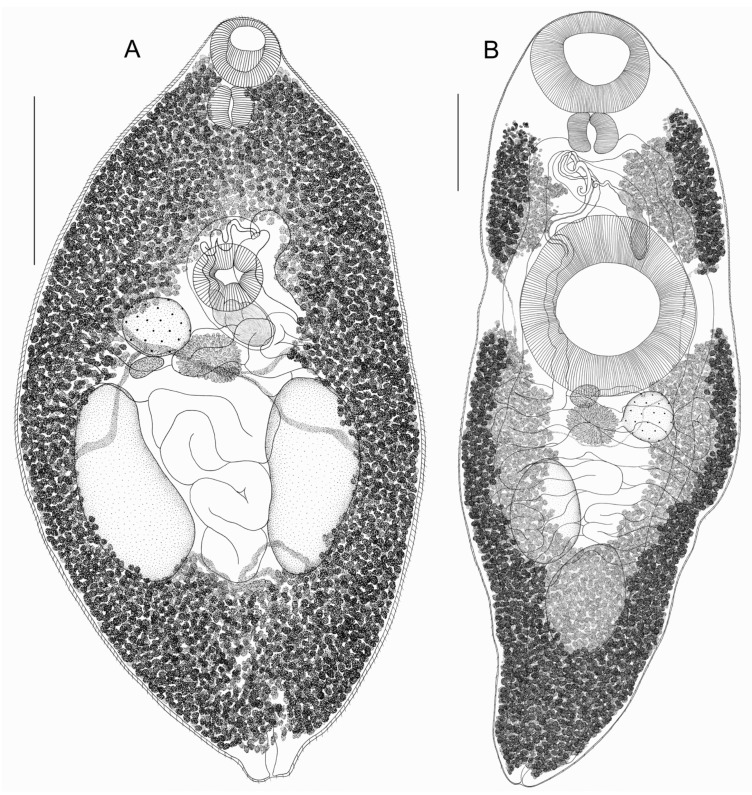
(**A**) General morphology of *Skrjabinoplagiorchis polonicus* ex *Apodemus uralensis*, ventral view; (**B**) general morphology of *Rubenstrema exasperatum* ex *Sorex araneus*; scale bars = 0.5 mm.

*Skrjabinoplagiorchis polonicus* (Sołtys, 1957) (Figure 2A)

Host: *Apodemus uralensis*.

Geographical locality: Novenkiy cordon, Mordovia Nature Reserve (Republic of Mordovia, 54.708200° N, 43.211900° E).

Prevalence: in three examined mice.

Intensity: 1–5 per infected rodent.

Mean intensity: 3.0.

Availability: GenBank No PX279693.

Accession numbers in collection of IEVB RAS: No 2202–2205.

General description of *Skrjabinoplagiorchis polonicus* (based on eight specimens, measurements are given in Table 1): Body oval, tapering towards anterior and posterior ends. Entire body covered with spines. Oral sucker rounded, subterminal, approximately equal to ventral sucker. Ventral sucker preequatorial. Pharynx large. Prepharynx, esophagus, and intestinal branches not visible due to densely located vitelline follicles. Testes large, longitudinally oval, entire; located symmetrically, postequatorially, or slightly shifted posteriorly. Cirrus wide, saccular, C-shaped, lies medially; located at level of ventral sucker and covered by it. Seminal vesicle clearly visible in cirrus sac. Genital pore somewhat submedial, located at anterior edge of ventral sucker. Ovary entire, rounded, located submedially at posterolateral edge of ventral sucker in front of right testis. Mehlis’ gland about same size as ovary, lies behind ventral sucker at posterolateral edge of ovary. Seminal receptacle small and pear-shaped, lies at posterior edge of ovary. Laurer’s canal not visible. Vitellarium well-developed, consists of numerous small rounded, pear-shaped or irregularly shaped follicles. Wide lateral fields of vitelline glands start from oral sucker and extend to posterior body end, completely covering intestinal branches. In anterior body part behind pharynx, and in posterior body part behind testes, vitelline fields merge medially on dorsal and ventral body sides. Main vitelline ducts merge along median line in front of testes at posterior edge of Mehlis’ gland, forming vitelline reservoir. Uterine loops pass between testes and occupy entire space between them, ventral sucker, and ovary. As a rule, uterine loops do not go beyond level of testes. Terminal uterus section has clearly defined metraterm. Eggs large and numerous, with golden-brown operculum. Excretory pore terminal.

Superfamily Plagiorchioidea Lühe, 1901

Family Plagiorchiidae Lühe, 1901

Subfamily Omphalometrinae Looss, 1899

Genus *Rubenstrema* Dollfus, 1949

*Rubenstrema exasperatum* (Rudolphi, 1819) (Figure 2B)

Host: *Sorex araneus*.

Geographical locality: Novenkiy cordon, Mordovia Nature Reserve (Republic of Mordovia, 54.707800° N, 43.213500° E).

Prevalence: in three examined shrews.

Intensity: 3–11 per infected shrews.

Mean intensity: 6.3.

Availability: GenBank No PX279692.

Accession numbers in collection of IEVB RAS: No 2220–2224, 2231–2233.

General description of *Rubenstrema exasperatum* (based on 18 specimens, measurements are given in Table 2): Body elongated–oval, rounded at anterior end and strongly narrowed posteriorly. Entire body, except for posterior end, covered with spines. Oral sucker subterminal, significantly smaller than ventral sucker. Ventral sucker preequatorial. Pharynx large. Prepharynx and esophagus not visible. Intestinal branches long and extend to hind body. Testes oval, entire, located diagonally postequatorially. Cirrus sac curved, elongated, located at anterior edge of ventral sucker. Distal end of cirrus sac usually hook-shaped. Proximal part of cirrus sac is occupied by seminal vesicle. Cirrus unarmed. Genital pore submedial, located behind pharynx. Ovary entire, rounded, located submedially at posterolateral edge of ventral sucker. Mehlis’ gland approximately equal to ovary, lies behind ventral sucker almost medially. Seminal receptacle small, pear-shaped, lies at posterior edge of ventral sucker and partially covered by it. Seminal receptacle and Mehlis’ gland located at level of ovary, on left side. Laurer’s canal lies at posterior edge of ventral sucker. Vitellarium well developed, consists of numerous small rounded, pear-shaped or irregularly shaped follicles. On each body side at level of ventral sucker, vitelline fields divided by certain interval into two groups. Anterolateral group of vitelline fields begins at level of pharynx and extends to level of anterior third of ventral sucker. Posterior lateral group of vitelline fields begins at level of posterior third of ventral sucker and reaches hind body. This group of vitelline fields merges on dorsal body side, from level of posterior testis to hind body, on ventral side—behind testes. Anterolateral groups of vitelline fields never merge. Main vitelline ducts merge along median line at posterior edge of Mehlis’ gland, forming vitelline reservoir. Uterine loops fill entire space from ventral sucker to posterior testis. One of uterine loops passes between testes. Distal part of uterus has well-defined metraterm. Uterine loops never extend beyond level of testes. Eggs large, numerous, with golden-brown operculum. Excretory pore terminal.

**Table 2:** Measurements of *Rubenstrema exasperatum* from this study and previous descriptions.

Characters	Our Study	Braun [25]	Dollfus [14]	Pojmanska [26]	Shaldybin [27]	Edelenyi [28]	Andreyko [24]	Genov [8]	Sharpilo, Iskova [1]
Host	*Sorex araneus*	*Neomys fodiens*	*Neomys fodiens*	*Sorex araneus*	*Sorex araneus, S. minutus, Neomys fodiens*	*N. anomalus*	*Sorex araneus*	*Neomys anomalus*	*Sorex araneus*
Locality	Mordovia	Austria	France	Poland	Mordovia	Hungary	Moldova	Bulgaria	Ukraine
Body length	2.861–4.684 (3.578)	4.0	4.6	2.03–3.18	2.0–4.5	3.27	3.1–3.8 (3.6)	4.02–6.05	2.5–5.1
Body width	0.886–1.722 (1.201)	1.4	1.3	0.70–0.99	0.850–1.275	0.84	1.98–2.24 (2.1)	1.150–1.809	1.1–1.7
Oral sucker	0.364–0.827 × 0.391–0.782 (0.509 × 0.521)	0.469 × 0.573	0.53 ^1^	0.29–0.43 × 0.39–0.44	0.221–0.476 ^1^	0.42 ^1^	0.43–0.59 (0.58)	0.543–0.643 × 0.603–0.703	0.45–0.60 × 0.50–0.70
Pharynx	0.146–0.273 × 0.164–0.273 (0.215 × 0.216)	0.26 × 0.27	0.22 × 0.19	0.16–0.19 × 0.13–0.16	0.136–0.221 ^1^	–	0.26–0.31 (0.28) ^1^	0.201–0.241 × 0.221–0.241	0.22–0.41 ^1^
Ventral sucker	0.655–1.018 × 0.618–0.936 (0.759 × 0.717)	0.80 ^1^	0.84 ^1^	0.47–0.63 × 0.46–0.60	0.510–0.846 ^1^	0.56 ^1^	0.793–1.050 (0.985)	0.884–0.944 × 0.924–0.964	0.60–1.10 × 0.71–1.00
Anterior testis	0.318–0.591 × 0.218–0.409 (0.477 × 0.310)	–	0.54 × 0.26	0.31–0.40 × 0.16–0.28	0.425–0.680 × 0.255–0.340	0.141	0.569–0.840 × 0.462–0.735 (0.840 × 0.735)	0.462–0.824 × 0.361–0.402	0.22–0.66 × 0.22–0.52
Posterior testis	0.364–0.682 × 0.218–0.455 (0.502 × 0.329)	–	0.56 × 0.28	0.38–0.53 × 0.20–0.32	0.561–0.731 × 0.272–0.306		0.420–1.008 × 0.493–0.798 (0.567 × 0.746)	0.683–0.864 × 0.281–0.462	0.33–0.82 × 0.16–0.44
Cirrus sac	0.455–0.709 × 0.127–0.209 (0.582 × 0.162)	–	–	0.25–0.42 × 0.09–0.14	0.425–0.765 × 0.136–0.255	–	0.672–0.810 (0.785)	0.603–0.763 × 0.120–0.261	0.37–0.70 × 0.14–0.27
Ovary	0.182–0.318 × 0.173–0.282 (0.229 × 0.232)	0.28 ^1^	0.30 × 0.21	0.16 × 0.20	0.170–0.272 ^1^	0.14 ^1^	0.210–0.418 × 0.315–0.441 (0.418 × 0.429)	0.261–0.321 × 0.241–0.321	0.18–0.33 × 0.22–0.33
Eggs	0.058–0.065 × 0.024–0.032 (0.061 × 0.027)	0.059 × 0.032	0.067–0.071 × 0.031–0.037	0.041–0.043 × 0.016–0.020	0.057 × 0.027	0.083 × 0.033	0.063–0.068 × 0.023–0.025	0.065–0.069 × 0.024–0.036	0.055–0.066 × 0.027–0.033

Note: ^1^—diameter; mean values are given in parentheses.

## 4. Discussion

In this study, we analyzed morphological and morphometric features of two omphalometrid species, *Skrjabinoplagiorchis polonicus* and *Rubenstrema exasperatum*, parasitizing small mammals in the Middle Volga region (European Russia), and obtained new molecular phylogenetic data on these trematodes. We also present a complete description of *S. polonicus* from the herb field mouse, *Apodemus uralensis*. We have confirmed the taxonomic affiliation of this species within the subfamily Omphalometrinae, which previously included only parasites of Eulipotyphla [15,16].

According to the results of our study, both examined species belong to the clade Omphalometrinae. In this context, *R. exasperatum* clusters with other representatives of the genus, while *S. polonicus* diverges earlier than other members of this subclade, occupying the most basal position among the Omphalometrinae. The overall arrangement of the remaining clades largely corresponds to previous studies (see, for example, Müller et al. [29]). Only certain subclades show variation, which can be explained by differences in the sets of sequences used. However, it is more likely that we have once again arrived at the previously expressed conclusion that a thorough revision of the phylogenetic relationships of several trematode families—Plagiorchiidae, Glypthelmintidae, Leptophallidae, Brachicoeliidae, etc.—is required simultaneously.

Before our study, the genus *Skrjabinoplagiorchis* was assigned to the subfamily Plagiorchiinae [1]. Representatives of the subfamilies Plagiorchiinae and Omphalometrinae are quite similar in both morphological and morphometric features. Exceptions include certain morphometric characteristics, such as the length of the uterus and the size of the eggs. As a rule, the uterus in plagiorchiids is long and extends into the post-testicular space, often reaching the posterior extremity of the body. Trematodes of the subfamily Plagiorchiinae produce relatively small eggs: their length does not exceed 0.050 mm, and their width is 0.029 mm [1]. In contrast, the subfamily Omphalometrinae includes trematodes whose uterus never extends into the post-testicular space, and their eggs are relatively large, reaching 0.071 mm in length and 0.037 mm in width [15]. In the studied specimens of *S. polonicus* and *R. exasperatum*, the uterus does not extend beyond the level of the testes, and the eggs are relatively large (Figure 2, Table 1 and Table 2). Based on these morphological features, we classify *S. polonicus* as a member of the Omphalometrinae subfamily. The molecular data obtained also support this conclusion (Figure 1). The minimal genetic differences between plagiorchiids and omphalometrids (Figure 1), along with the fact that the host range of the latter group is no longer limited to Eulipotyphla, support the suggestion of Tkach et al. [16] that omphalometrids form a subfamily within the family Plagiorchiidae.

All previously investigated adult digeneans of the Omphalometrinae are known as parasites of insectivores. However, the examined species of the genus *Skrjabinoplagiorchis* parasitizes rodents. It is probable that a shift of definitive host occurred during the differentiation of *Skrjabinoplagiorchis*. This transition may be attributed to the similar diet of Eulipotyphla and Rodentia, which both include invertebrates that serve as intermediate hosts for the parasite.

We observed some morphological variability in the sizes of the body, suckers, and reproductive organs in *S. polonicus* and *R. exasperatum*. Only the egg sizes remained relatively constant in both species. Generally, the characteristics of all studied specimens of both omphalometrids were in good agreement with previous descriptions (Table 1 and Table 2). In *R. exasperatum*, the following characteristic features were consistently present: division of the vitelline fields at the level of the middle third of the ventral sucker, the location of the cirrus sac in the space between the pharynx and the ventral sucker, the diagonal arrangement of the testes, and the position of the posterior edge of the uterus at the level of the posterior testis. However, previous studies do not indicate whether the vitelline fields merge ventrally along the midline. In most cases, it is indicated that the vitelline fields merge medially behind the testes, but it is impossible to determine from the drawings at what level and on which side of the body the vitellarium merges [1,8,14,24,25,26,27,28]. In the *R. exasperatum* specimens we examined, the vitelline glands merge along the midline at the posterior part of the body, both ventrally and dorsally (Figure 2B).

The studied specimens of *S. polonicus* also consistently exhibited characteristic features, including the symmetrical arrangement of longitudinally oval testes, wide vitelline fields extending from the oral sucker to the hind body and merging along the midline in both the anterior and posterior parts of the body, and uterine loops that do not extend beyond the level of the testes. However, we noted some differences in the topology of the inner organs of the specimens we studied compared to those described earlier [1,2,4,8,10,11,24]. Thus, Sharpilo and Iskova [1] specified that in *S. polonicus*, the cirrus sac is usually located at the anterior edge of the ventral sucker, and its base does not extend beyond the posterior edge of the sucker. This was also noted by Petrov and Merkusheva [4], Kadenatsii [12], Varenov [9], and Semenova [10]. Tkach [17] similarly mentioned the position of the cirrus sac in front of the ventral sucker. In contrast, in the *S. polonicus* specimens we examined, the cirrus sac is located in the area of the ventral sucker and is covered by it, with the base of the cirrus sac extending beyond the posterior edge of the ventral sucker. This feature was also noted by Andreyko [7], Genov [8], and Fedorov [11]. At the same time, Andreyko [7] and Genov [8] observed variability in the location of the cirrus sac.

Most studies indicate that the vitelline fields of *S. polonicus* merge in the anterior and posterior regions of the body [1,4,7,9,12,17]. Andreyko [7] and Sharpilo and Iskova [1] observed that this merging occurs on the dorsal side of the body. In the specimens of *S. polonicus* we examined, the vitelline fields merged in both the anterior and posterior regions, occurring dorsally as well as ventrally.

Andreyko [7] and Genov [8] noted that the uterine loops may slightly protrude beyond the posterior edge of the testes; however, in most cases, this does not occur. In our study, the uterine loops of *S. polonicus* did not extend beyond the posterior edge of the testes.

Previous descriptions of *S. polonicus* revealed variability in the position of the ovary. Thus, several authors reported that the ovary is located at the level of either the anterior or posterolateral edge of the ventral sucker, with the sucker partially overlapping the ovary [1,9,10]. According to Andreyko [7], Fedorov [11], Genov [8], Petrov and Merkusheva [4], and Kadenatsii [12], the ovary is consistently located above the right testis, a finding corroborated by our study (Figure 2A).

Sharpilo and Iskova [1] and Tkach [17] indicate that the seminal receptacle in *S. polonicus* is either not expressed or absent. Other authors have not reported the presence/absence of the seminal receptacle in this species. In *Skrjabinoplagiorchis* specimens we studied, the seminal receptacle is present and located behind the ovary.

Thus, *S. polonicus* exhibits significant morphological variability, a phenomenon previously noted by Andreyko [7] and Genov [8]. This is especially true for such features as the position of the cirrus sac and ovary. Therefore, it is important to consider the high variability of these morphological features when identifying trematodes of this species. Furthermore, *S. polonicus* is morphologically very similar to *Plagiorchis arvicolae*, which also parasitizes rodents [1,7]. Consequently, researchers may misidentify *S. polonicus* as *P. arvicolae* and vice versa. Our study also revealed that the dimensions of the body, suckers, and reproductive organs of *S. polonicus* may overlap with the corresponding characteristics of *P. arvicolae* [1]. Sharpilo and Iskova [1] indicate that the main differences between *S. polonicus* and *P. arvicolae* are their localization within the host and the size of the eggs. We have reached a similar conclusion. Additionally, we propose several more distinguishing morphological features, namely the position of the uterus and vitelline fields, as well as the position and shape of the testes.

In summary, when identifying *S. polonicus* and *P. arvicolae*, the following distinguishing features should be considered:

(1) Position of the testes. In *S. polonicus*, the testes are usually located symmetrically or almost symmetrically; in *P. arvicolae*, the testes are consistently located somewhat diagonally;

(2) Shape of the testes. In *S. polonicus*, the testes are invariably longitudinally oval, whereas in *P. arvicolae*, the testes may be oval, round, or circular;

(3) Position of the uterus. In *S. polonicus*, the uterus does not extend into the post-testicular space; in *P. arvicolae*, the uterus significantly extends beyond the level of the posterior testis;

(4) Merging of the vitelline fields. In *S. polonicus*, the vitelline fields merge medially in the anterior and posterior regions of the body, both dorsally and ventrally; in *P. arvicolae*, the vitelline fields predominantly merge only in the anterior part of the body and exclusively dorsally;

(5) Size of eggs. In *S. polonicus*, the egg size is 0.050–0.070 × 0.020–0.034 mm (Table 1); in *P. arvicolae*—0.034–0.043 × 0.017–0.022 mm [1];

(6) Site within the host. Adult flukes of *S. polonicus* are primarily localized in the liver ducts and less frequently found in the small intestine, where they end up after the host’s death [1]; adult individuals of *P. arvicolae* are exclusively localized in the small intestine of the host.

## 5. Conclusions

In this study, we present morphological and morphometric descriptions of two trematode species, *Skrjabinoplagiorchis polonicus* and *Rubenstrema exasperatum*, from small mammals in Russia. We also provide the first molecular phylogenetic data on *S. polonicus*. According to our findings, we propose the transfer of the genus *Skrjabinoplagiorchis* to the subfamily Omphalometrinae (Plagiorchiidae *s. str.*). The results of our study support the suggestion of Tkach et al. [16] that omphalometrids form a subfamily within Plagiorchididae. Being a parasite of rodents of the family Muridae, *S. polonicus* occupies a basal position relative to other omphalometrid species parasitizing insectivores. This may indicate a probable host shift from Eulipotyphla to Rodentia during the evolution of this digenean lineage.

## Figures and Tables

**Table 1:** Measurements of *Skrjabinoplagiorchis polonicus* from this study and previous descriptions.

Characters	Our Study	Soltys [2]	Petrov, Merkusheva [4]	Semenova [10]	Fedorov [11]	Genov [8]	Andreyko [24]	Sharpilo, Iskova [1]
Host	*Apodemus uralensis*	*Microtus agrestis*	*Apodemus uralensis*	*Apodemus flavicollis*	*Ondatra zibethicus*	*Ondatra zibethicus*	*Ondatra zibethicus*	*Arvicola amphibius*
Locality	Mordovia, Russia	Poland	Belarus	Volgograd Oblast, Russia	West Siberia, Russia	Bulgaria	Moldova	Ukraine
Body length	2.28–3.32 (2.89)	1.22–2.50	4.89–5.73	3.02–3.40	1.47–3.41	1.68–3.16	2.43–3.78 (3.64)	1.7–2.0
Body width	1.19–1.87 (1.56)	1.15–1.56	1.95–2.43	1.69–2.24	0.56–1.19	0.86–2.00	1.23–2.43 (1.87)	1.2
Oral sucker	0.191–0.336 × 0.209–0.355 (0.257 × 0.277)	0.31–0.35 ^1^	0.23–0.42 × 0.32–0.47	0.23–0.42 × 0.36–0.46	0.131–0.245 × 0.120–0.250	0.211–0.358 ^1^	0.253–0.311 × 0.243–0.315 (0.253 × 0.308)	0.22–0.24 × 0.24–0.27
Pharynx	0.127–0.203 × 0.136–0.215 (0.164 × 0.166)	0.18–0.24 ^1^	0.18–0.28 × 0.23–0.37	0.15–0.42 × 0.16–0.23	0.114–0.157 × 0.102–0.186	0.147–0.211 × 0.126–0.274	0.162–0.242 × 0.149–0.189 (0.198 × 0.187)	0.16 ^1^
Ventral sucker	0.200–0.323 × 0.200–0.318 (0.242 × 0.251)	0.31–0.36 ^1^	0.32–0.44 ^1^	0.33–0.42 ^1^	0.145–0.261 ^1^	–	0.266–0.365 × 0.264–0.372 (0.297 × 0.332)	0.24–0.25 × 0.24–0.27
Right testis	0.455–0.736 × 0.255–0.409 (0.628 × 0.333)	0.65–0.89 × 0.32–0.41	1.4–2.2 × 0.52–0.84	0.43–0.63 × 0.33–0.37	0.125–0.437 × 0.125–0.269	0.375–0.880 × 0.189–0.422	0.660–1.055 × 0.352–0.715 (1.045 × 0.715)	0.51–0.68 × 0.30–0.46
Left testis	0.409–0.636 × 0.264–0.427 (0.576 × 0.329)	0.65–0.91 × 0.32–0.41	1.30–1.96 × 0.60–0.79	0.43–0.67 × 0.33–0.37	0.125–0.456 × 0.068–0.226	0. 379–0.759 × 0.183–0.443	0.675–1.215 × 0.385–0.721 (1.215 × 0.719)	
Cirrus sac	0.500–0.655 × 0.109–0.159 (0.588 × 0.138)	0.49–0.51	–	–	0.226–0.780 × 0.047–0.078	0.892–0.937 × 0.095–0.098	1.35–1.65 (1.39)	–
Ovary	0.164–0.346 × 0.195–0.318 (0.283 × 0.251)	0.255–0.393 ^1^	0.35–0.48 ^1^	0.34–0.46 ^1^	0.113–0.250 × 0.068–0.250	0.168–0.379 × 0.066–0.070	0.220–0.374 × 0.165–0.379 (0.325 × 0.374)	0.16–0.23 × 0.17–0.33
Eggs	0.052–0.057 × 0.026–0.028 (0.054 × 0.027)	0.059–0.066 × 0.020–0.033	0.055–0.065 × 0.027–0.034	0.050–0.061 × 0.022–0.027	0.057 × 0.034	0.062–0.070 × 0.024–0.033	0.066–0.069 × 0.033	0.050–0.065 × 0.027–0.030

Note: ^1^—diameter; mean values are given in parentheses.

## Data Availability

GenBank numbers are given in the relevant section of the manuscript. Any other data is available after a reasonable request.

## Supplementary Materials

The following supporting information can be downloaded at: https://www.mdpi.com/article/10.3390/biology14101423/s1, Table S1: Sequences used in molecular phylogenetic analysis [14,17,29,30,31,32,33,34,35,36,37,38,39,40,41,42,43,44,45,46].

## Abbreviations

The following abbreviations are used in this manuscript:

IEVB	Institute of Ecology of the Volga River Basin
RAS	Russian Academy of Sciences
subfam.	Subfamily
